# Progress and Prospects of Research on the Impact of Mental Health of Youth Sailors—A Bibliometric-Based Analysis

**DOI:** 10.3390/healthcare13060608

**Published:** 2025-03-11

**Authors:** Milena Lachowicz, Yang Xing, Tomasz Chamera

**Affiliations:** Department of Social Sciences, Gdańsk University of Physical Education and Sport, 80-336 Gdańsk, Poland

**Keywords:** youth athletes, youth sailors, mental health, well-being, sports psychology

## Abstract

**Background:** The mental health of youth sailors has garnered increasing attention from both coaches and researchers, as evidenced by the growing appearance of related keywords in scientific literature. Despite this rising interest, no studies have yet specifically focused on the mental health of this population. **Methods:** This study conducted a bibliometric analysis of 315 articles retrieved from the Web of Science database. These articles were analyzed to identify trends, influential authors, institutions, and regions in the field of youth sailor mental health. **Results:** The analysis yielded several key findings: (1) Depression, anxiety, and mental health disorders are the primary areas of focus in the literature on youth sailors’ mental health; (2) Rosemary Purcell is identified as the most influential author in this domain; (3) the University of Melbourne, Orygen, and Deakin University are the top three contributing institutions; (4) Australia, the USA, Canada, China, and England are the five most prominent regions involved in this research. **Conclusions:** This study provides a comprehensive overview of current research on the mental health of young sailors. By emphasising the most influential contributors and research trends, it aims to raise awareness amongst coaches and researchers, eventually supporting efforts to improve the mental health of young sailors.

## 1. Introduction

Sport psychology emerged as a formal discipline in the United States during the 1970s [1,2,3] with early research primarily focusing on psychological assessment methods and the validity of these techniques in evaluating athletes. Most of this work was published in journals such as the *Journal of Sport Psychology* [4]. Over time, as advancements in science and societal understanding of mental health grew, the application of psychology expanded beyond mere assessment, drawing attention to mental health issues among youth [5,6]. Youth mental health has become a significant component of the global disease burden [7,8,9]. In countries like the United States, the prevalence of mental health disorders among youth has reached 22% [10,11,12], with many issues persisting into adulthood.

Recent estimates suggest that mental disorders accounted for a significant proportion of the global disease burden in 2019, contributing to millions of disability-adjusted life years lost. The economic impact of mental health conditions has been estimated at around USD 1 Trillion annually, with long-term projections reaching several trillion dollars. Individuals with mental disorders also had a 2.2 times higher chance of premature death, and therefore stronger interventions and policies in the area of mental health need to be implemented [13].

Research indicates that youths involved in sports tend to report higher overall life satisfaction and lower levels of anxiety and depression compared to their non-participating peers [14]. However, despite the generally positive effects of sports, young athletes still face significant mental health challenges [15,16]. In sports requiring sustained high performance, such as sailing, athletes are at greater risk of experiencing mental health issues due to the prolonged stress and intense competition [17,18]. The physical and psychological demands of long-term training may contribute to the development of depression and other mental health disorders [19].

Since the onset of the COVID-19 pandemic in 2020, mental health issues among athletes have become a popular topic in academic discourse [20]. During this period, youth sailors, despite maintaining their training, experienced a marked increase in issues such as substance abuse, sleep disorders, and emotional disturbances [21]. This rise in mental health problems has garnered increased attention from coaches and parents, as well as a growing awareness of the difficulties youth sailors face in their training environments. Unlike the immediate support they might receive following a sports-related loss, young sailors often struggle to find effective coping mechanisms for mental health issues. Social stigma surrounding mental healthcare exacerbates the problem, with many youths reluctant to seek help due to concerns about their self-esteem. As a result, addressing mental health concerns and providing psychological support in sailing training has become a pressing need [22].

Over the past decade, there has been increasing global awareness of youth athlete mental health, with research spanning psychological resilience, emotional regulation, and competitive stress [23,24]. The mental health needs of young athletes are shaped by sport-specific stressors, yet much of the literature focuses on team sports and endurance training, leaving gaps in understanding the unique psychological burdens faced by youth sailors. Unlike other athletes, sailors must manage cognitive demands in dynamic environments, navigate high-stakes decisions, and cope with isolation, overtraining, and sleep disruptions [8]. Given the psychological vulnerabilities associated with prolonged exposure to environmental stressors, further research is required to explore the mental resilience and coping mechanisms of youth sailors. This study conducts a bibliometric analysis to map existing research trends and highlight critical knowledge gaps, offering insights into future research directions. This study addresses the mental health of youth sailors by employing bibliometric analysis, a quantitative method that has been widely used across various academic fields to examine trends and knowledge structures [25]. To the best of our knowledge, this is the first study to apply bibliometric methods to analyze the mental health of youth sailors, which is a critical yet underexplored area of research. The main objectives of this study are as follows: (1) To identify key research terms and hotspots in this field, allowing us to analyze and predict future research trends; (2) to determine the most influential authors contributing to this research area, which may help clarify the leading voices and perspectives within the field; (3) to emphasize the countries that have made the most important contributions to this body of research, providing a geographical context to the findings; (4) to identify the key journals that publish articles on this important topic, displaying where most of the academic discourse occurs; and (5) to explore the number of articles published in this field over the past decade, offering insight into how research interest has evolved over time.

Despite increasing focus on youth athletes’ psychological health, few systematic bibliometric studies have focused on studies on research trends, leading authors, and future areas of studies. Recent studies have focused on general youth athletes, while studies on individual youth athletic subpopulations, such as sailors, are lacking. Sailing is physically and psychologically demanding, and therefore, understanding sailors’ psychological health is crucial. We aim to fill this gap in the literature by applying a bibliometric approach to analyze the impact of mental health on youth sailors, providing insights on future directions and leading authors in such studies. Moreover, unlike systematic reviews and meta-analyses, where evidence is synthesized and interventions’ effects are weighed, bibliometric analysis presents an overview of research landscapes on a macroscale, revealing influential authors, institutions, and subject trends. The methodology is the most valuable in emerging disciplines where quantitative structures and trends of knowledge have to be mapped in order to guide future studies.

This study aims to provide a comprehensive overview of the current state of research on youth sailors’ mental health, offering valuable insights for future academic development and emphasizing the importance of psychological support throughout sailors’ training. By collating and analyzing these varied aspects of academic literature, we hope to not only inform practitioners and policymakers but also to raise awareness about the unique mental health challenges faced by this group, eventually contributing to the enhancement of support systems available to young sailors.

## 2. Materials and Method

### 2.1. Design

This study employs a descriptive bibliometric analysis to examine the field of sailors’ mental health. As a quantitative approach, bibliometric analysis enables the identification of key variables such as research output [26], country of origin, research organizations, authorship patterns, keywords, and title trends. The methodology allows for the exploration of the knowledge structure and emerging trends in the field of athletes’ mental health, particularly focusing on sailors. The bibliometric analysis was conducted using two specialized software packages, VOSviewer 1.6.20 and CiteSpace 6.3.1, both of which are widely recognized tools for mapping and visualizing knowledge domains in academic research [27].

### 2.2. Data Retrieval

Data for this study were obtained from the Web of Science (WOS) Core Collection database, a premier source for bibliometric research [28,29,30,31]. The search focused on two citation indexes, SCI-EXPANDED and SSCI, ensuring comprehensive coverage of relevant publications. The analysis spanned a period of ten years, from 1 January 2014 to 24 July 2024. The WOS database is employed in this research due to comprehensive top-journal coverage and strict criteria for including journals. Compared to other databases, WoS assures quality, peer-reviewed content and extensive citation metrics, vital for citation analysis and data collection. Despite access to other databases, including PubMed and Scopus, and their studies, WOS is utilized to maintain data collection and citation consistency.

A series of search terms related to mental health in young athletes, specifically sailors, was employed. These terms included “youth mental health sport”, “youth mental health sailors”, and “youth mental health athletes”. The keywords were established through initial searches, specialist consultation, and preceding literature in youth sailing and mental health. Refining strategies included applying Boolean search operators and truncation to capture broadly while minimizing irrelevant data. For example, (‘youth mental health’ AND ‘sailing’ OR ‘aquatic sports’) was used to identify studies in youth sailors while excluding broader classes of athletes. The search yielded a total of 1296 articles published between 2014 and 2024. These articles were classified into various document types, including 1084 original research articles, 165 review articles, 39 early access papers, 24 meeting abstracts, 19 editorial materials, 7 retracted publications, 3 letters, 2 proceedings papers, and 1 correction. Ethical approval was not required for this study as all data were obtained from publicly available sources. The details of the data retrieval process are summarized in Table 1.

### 2.3. Data Cleaning and Selection

Given the nature of bibliometric data retrieval, it is common for the dataset to include irrelevant or duplicate records. To improve the reliability and accuracy of the data, a systematic data cleaning process was implemented, abbreviated as DEAN (Duplicates, Errors, Alias, and Noises). To ensure integrity in data, the DEAN methodology (Duplicates, Errors, Alias, Noises) was utilized. The duplicates were filtered using the inbuilt deduplication function in CiteSpace. The authors’ names were normalized by grouping variations (i.e., ‘J. Smith’ and ‘John Smith’). Spurious data with no relation to mental health were filtered to maintain dataset integrity. This process involved the removal of duplicate entries, correction of errors in metadata, consolidation of author aliases, and exclusion of irrelevant or noisy data. After the cleaning process, 315 articles were selected for further analysis from the original 1296 records. The final selection of articles, categorized by document type, is presented in Table 2.

### 2.4. Data Analysis

The final dataset of 315 articles was subjected to detailed bibliometric analysis. Three main methods were employed: (a) Hirsch Index (h-index) was calculated to identify the most influential papers in terms of citations. (b) Bradford’s Law was applied to classify journals into core and peripheral sources, helping determine the most impactful publications in the field [32]. (c) VOS viewer was utilized for keyword co-occurrence analysis, allowing for the identification of frequently occurring terms and the construction of keyword clusters to map research trends [33,34].

**Figure 1:**
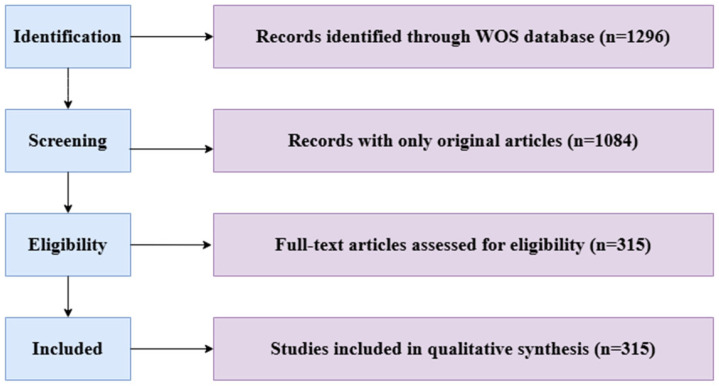
Database acquisition flowchart for the visualization study.

The data analysis was aimed at elucidating the major contributors, core journals, and key research themes in the domain of sailors’ mental health. A visualization of the article selection process is provided in Figure 1.

## 3. Results

A. Publication Trends

The bibliometric data illustrate heightened activity in youth athletic mental health studies in recent decades. The citation pattern and leading authors point to hubs of quality in mental health in sports studies, who might be future collaborative opportunities for clinical studies. Furthermore, the keyword pattern illustrates heightened focus on disorders including anxiety and depression, confirming the urgent imperative for health interventions in youth athletes. These data provide evidence to health policymakers and sporting institutions on priority areas in athletic mental health studies.

After data cleaning, 315 papers authored by 1468 individuals from 716 institutions across 65 countries, and published in 143 journals, were included in this study. These papers cited a total of 13,864 references from 4857 journals. Notably, seven references were cited more than 20 times.

Calculations were based on Price’s Law formula [35]. The collected literature was analyzed. Where n(x) denotes the number of authors who published x articles, I = n_max_ is the number of authors who published the most articles, n_max_ = 20, N is the total number of authors, and m is the number of articles published by core authors with the least number of articles. Price’s Law suggests that the minimum number of publications by core authors in each field is m = 0.749 ∗ $\sqrt{n_{\max}}$ ≈ 3.35, so that more than three authors are positioned as core authors in the field. There are 25 core authors in the 315 studies with a total of 159 articles published, accounting for 50.48% of the total articles.

B. Periodical Production

**Figure 2:**
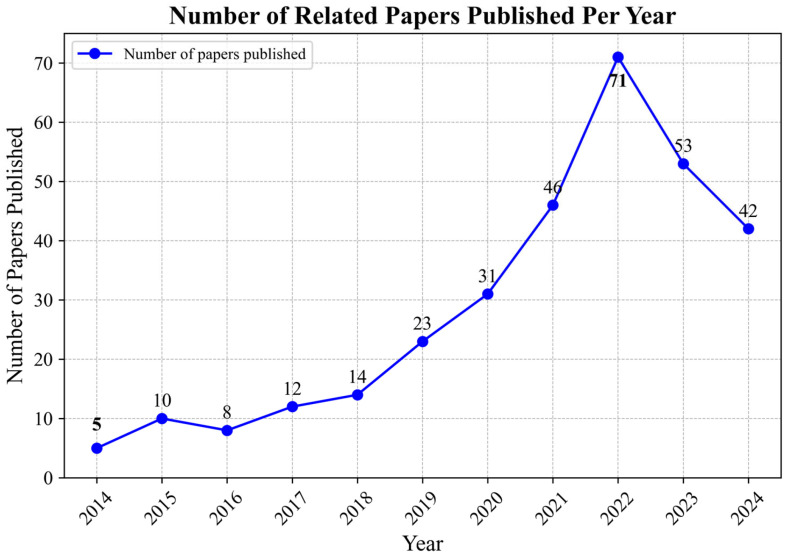
Number of related papers published per year.

From 2014 to July 2024, the number of publications on this topic experienced a remarkable surge, escalating from just 5 papers in 2014 to an impressive total of 42 papers in 2024. The year 2022 marked a peak in publication rate, with a striking 71 papers released, indicating a clear upward trend that has developed over the past decade (see Figure 2). The observed post-2020 increase in publications may have happened through increased focus on psychological problems in athletes in response to the COVID-19 pandemic, where psychological issues in athletes were given prominence. Policy shifts regarding welfare in sporting administration may have also influenced greater research focus. This consistent growth not only emphasizes the rising significance attributed to the mental health of athletes, particularly among sailors, but also suggests the increasing recognition of the challenges faced by these individuals in their competitive environments.

Utilizing Bradford’s Law [36], the academic journals were methodically categorized into three distinct zones based on the volume of articles published. The ratio of journals across these three zones approximates to 1:3:9, aligning well with the theoretical expectations posited by Bradford’s Law (see Table 3). The First Zone, comprising eight journals, is notable for contributing 84 articles throughout the decade, thereby representing the foundational sources in this area of research. The Second Zone featured 21 journals that collectively published 86 articles, while the expansive Third Zone consisted of 114 journals that generated 145 articles. This distribution not only emphasizes the important concentration of research outputs within a select group of core journals but also reflects the diverse range of studies emerging from a broader array of publications in the field of athlete mental health.

C. Country/Region Analysis

**Figure 3:**
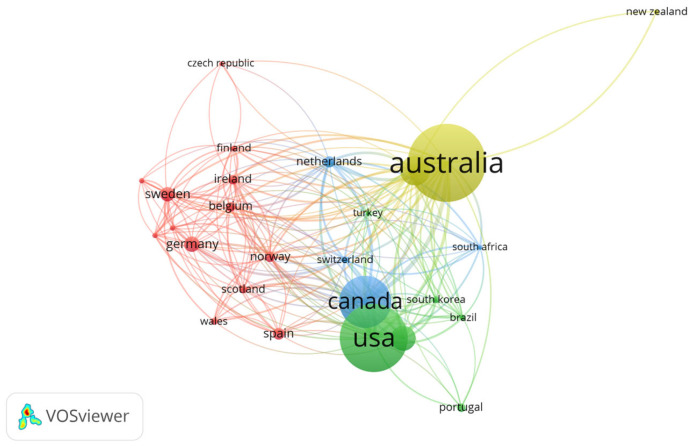
Number of papers published in the field in different countries.

The study identified 65 countries contributing to the literature on athletes’ mental health (Table 4). Australia led the field with 97 publications, followed by the USA (84 papers) and Canada (64 papers). In terms of total citations, Australia also ranked first with 3065 citations, while Canada had the highest average citations per article (35). Figure 3 illustrates the global distribution of research output, with Australia, the USA, and Canada being the largest contributors. The size of the circles in the figure represents the volume of publications, while the color groupings indicate the research associations between countries.

D. Research Organisations

**Figure 4:**
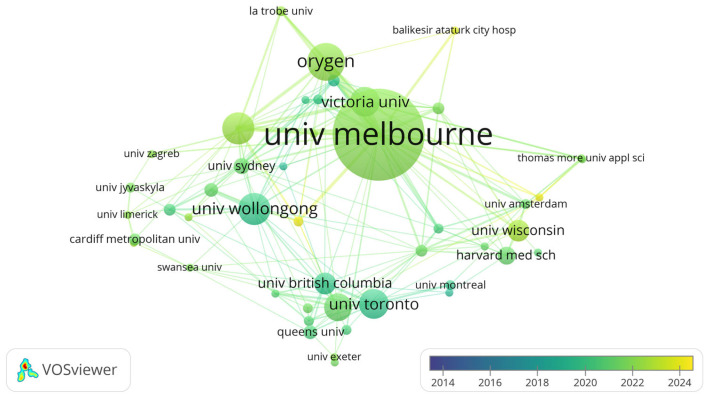
Initiation and impact of the research conducted by different organizations.

The 315 articles analyzed in this study were published by researchers from 716 institutions. The University of Melbourne led with 46 articles, significantly more than the second-highest contributor, Orygen, with 19 articles (Table 5). The University of Melbourne and the University of Wollongong both accumulated 1036 citations, although the latter had a much higher average citation per article (70.45). Figure 4 depicts the timeline of research contributions from these major institutions, with the University of Wollongong and the University of Toronto initiating research earlier than the University of Melbourne.

E. Author Contributions

**Figure 5:**
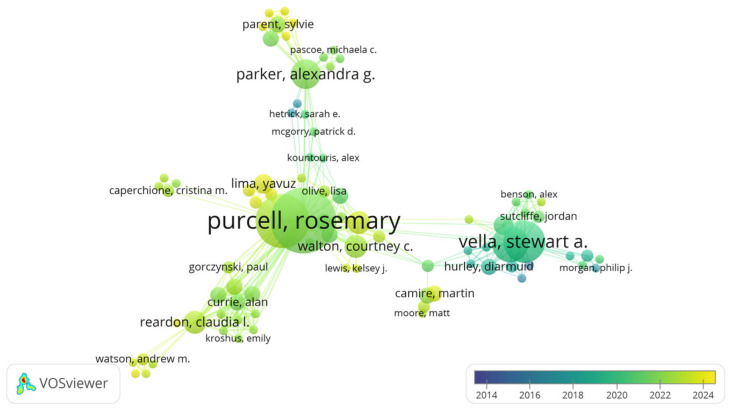
Authors’ contributions to research in different years.

Among the 1468 authors involved in this field, Purcell Rosemary led with 20 articles and 584 citations, making her the most prolific author in this research domain (Table 6). Reardon Claudia I., however, had the highest average citation per article, with 36.5. The analysis reveals that different authors have contributed significantly across various periods, as shown in Figure 5, with Purcell, Parker, and Vella representing key figures in distinct phases of research development.

F. Journal Analysis

Of the 143 journals included in this study, *Psychology of Sport and Exercise* and *Frontiers in Psychology* each published 17 articles. However, the journal with the highest total citations was the *International Journal of Research and Public Health*, with 505 citations and an average of 33.67 citations per article (Table 7). This journal’s high impact in the field underscores its central role in disseminating critical research on athletes’ mental health.

G. Keyword Analysis

**Figure 6:**
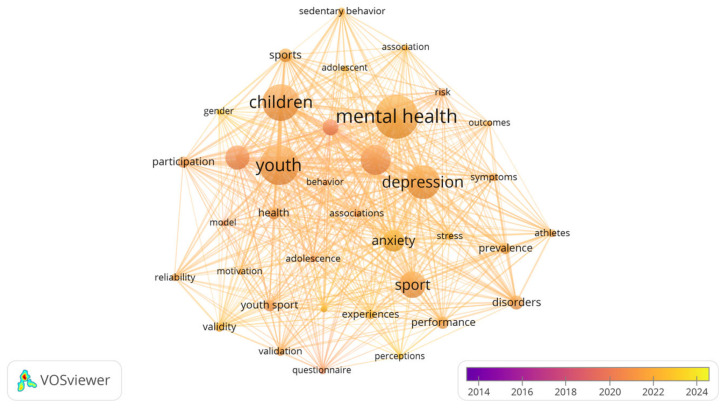
Year of appearance and impact of different keywords.

A total of 1433 keywords were identified across the 315 articles. The most frequently occurring keywords were Mental Health (145 times), Youth (89 times), and Physical Activity (89 times), reflecting the central themes in this field (Table 8). Negative mental health outcomes such as Depression (76 times), Anxiety (49 times), and Disorders (32 times) were also prevalent, highlighting key areas of concern in athlete mental health research. Figure 6 depicts the year of appearance and impact of different keywords.

## 4. Discussion

This study represents the first bibliometric analysis focusing on mental health research in athletes, particularly young athletes and aquatic athletes. The primary objective was to explore the progression and focus of literature related to the mental health of young athletes and aquatic athletes. Through a detailed analysis of the literature from the Web of Science (WOS), we identified key trends, influential authors, and major research institutions in this domain. A total of 315 articles, selected from an initial pool of 1296, were found to align with the theme. The number of publications has shown a consistent upward trend since 2014, peaking in 2022. Additionally, this study investigated the most active countries, frequently occurring keywords, top journals, and key contributing institutions. The findings of this bibliometric analysis identify increased global interest in youth athletes’ mental health [37], including endurance and top-level sports such as sailing. From a health perspective, this is in accordance with the imperative for psychological interventions in youth athletes to occur in their early phases. Research has established that incorporation of interventions for mental health in training regimens is capable of preventing youth athletes’ anxiety and depression. Future studies have to focus on evidence-based interventions for youth athletes’ mental health and their evaluation in preventing psychological disorders in youth athletes in the long run.

1. Increasing Interest in Mental Health of Athletes

The annual growth in publications emphasizes the increasing attention on the mental health of athletes, especially since 2014, when only five articles were published. This upward trend coincides with a broader global focus on the psychological well-being of athletes, influenced by the growing internationalization of sports and the recognition of mental health as a critical factor in athletic performance. As more international sporting events take place, researchers are expanding their focus beyond physical training and performance to address the psychological challenges athletes face [38], such as anxiety, depression, and the pressures of competition. This aligns with a developing body of work in sports physiology and psychology, where mental health is now recognized as a key area of concern, reflecting a major shift in how we understand the comprehensive nature of athletic performance.

2. Key Journals, Authors, and Institutions

Our analysis identified 143 journals contributing to this field, with *Frontiers in Psychology*, *Psychology of Sport and Exercise*, and the *International Journal of Environmental Research and Public Health* being the most prominent. The former two are dedicated to psychology, reflecting the interdisciplinary nature of mental health research in sports.

Rosemary Purcell from the University of Melbourne emerged as the most prolific author in this field, reinforcing the significant contributions from Australian researchers. Both the University of Melbourne and the University of Wollongong were among the most frequently cited institutions, highlighting Australia’s leadership in youth mental health and sports psychology, particularly with regard to aquatic athletes [39,40].

3. Emerging Research Trends and Gaps

On a broader scale, this study revealed an increasing global awareness of the psychological experiences of young athletes in different social and cultural contexts. Researchers have begun to focus on the micro-level factors influencing the development of youth athletes, including emotional regulation, mental resilience, and the impact of competitive pressures. Recent literature has identified an emergent world-wide interest in the psychosocial well-being of youths and specifically how youths experience the micro-level psychosocial stresses of sport participation [24]. Emotional regulation, resilience, and competitive anxiety have been identified as the major predictors of psychosocial welfare among youths and teenagers in sport [9]. This study however identifies that within the general move toward the psychosocial welfare of youths in sport, the psychosocial welfare of youths in sailing has been an understudied population within this literature.

Sailing involves a distinct set of physical, intellectual, and external challenges that set it apart from conventional sports. Sailing athletes typically do not experience the instant social support of fellow athletes found in the majority of sports; instead, they perform in individualistic or small-crew environments that enhance feelings of loneliness and decision fatigue that amplify psychological strain [41]. Sailing also necessitates the need for constant attention and speedy decision-making within changing environments that enhance the likelihood of cognitive fatigue and mental fatigue [8]. This study outlines that although general sport psychologist literature has grown in scope, sport-specific findings regarding sailing-associated psychological stresses are limited.

One area of existing literature that has been lacking is the limited study of evidence-supported interventions among youth sailors. Sleep hygiene programs, resilience training, and psychological support systems have been researched comprehensively within the area of mainstream team sports; however, it has not been established whether they are also effective within the area of marine endurance sports [42]. Considering that sleep disturbances and the syndrome of overtraining are frequent among sailors, future study needs to be dedicated toward the development of specific psychological interventions that treat such particular risk factors [43]. Also, economic and sponsorship anxiety emerge as additional sources of psychosocial burden among sailors that oppose the award-based funding schemes of college sports teams [44]. Athletic performance in sports teams is typically assessed collectively, yet sailing involves individual responsibility that goes beyond the sport and raises anxiety and psychological strain [45]. This study emphasizes the need for transdisciplinary collaborative efforts among sports psychologists, neuroscientists, and sailing sports organizations that bridge the space between the science of mental health and applied sailing interventions.

So, this study underscores the necessity of expanding mental health research beyond traditional athletic disciplines. By incorporating a sailing-specific perspective, future studies can develop holistic, evidence-based interventions that cater to youth sailors’ psychological resilience, cognitive workload, and emotional regulation strategies, ensuring long-term well-being in high-performance competitive environments. Similar to endurance and gymnasts, youth sailors experience prolonged stress through intensive training, competition pressure, and expected performance. As compared to shore-based sporting disciplines, sailors have to contend with extrinsic stressors in their environment, such as isolation and unpredictable weather, and thus psychological interventions have to be individualized [46].

Our keyword analysis uncovered frequent references to terms like “depression”, “anxiety”, and “disorders”, reflecting the significant mental health challenges young athletes face. Alarmingly, nearly 39.6% of youth athletes (aged 12–25) have experienced mental health disorders [47]. While efforts are being made to address these issues through coaching interventions and psychological counseling, stigma around mental health remains a major barrier [48,49]. Youth athletes often hesitate to seek help due to perceived negative attitudes toward therapy, limiting the effectiveness of mental health interventions.

4. Practical Implications and Future Research Directions

The findings of this study have practical implications for sports organizations, coaches, and families involved in youth athletics. Given the prevalence of mental health issues like anxiety and depression among young athletes, it is crucial to create a supportive environment that promotes mental well-being. Mental health literacy programs, such as those designed to improve resilience and reduce stigma, have shown promise in helping athletes manage stress and mental health challenges. However, further research is needed to explore the long-term effectiveness of such interventions, especially across different sports and cultural settings.

Furthermore, the sport environment offers untapped potential for improving mental health outcomes. Future research should investigate the development of targeted interventions tailored to specific athlete populations, such as aquatic athletes or those in high-performance programs. Studies could also explore how different social environments and team dynamics influence athletes’ psychological health [50,51].

5. Limitations and Conclusions

While this study provides a comprehensive bibliometric analysis of mental health research in athletes, it is not without limitations. The analysis is restricted to publications indexed in WOS, potentially omitting relevant studies from other databases. Additionally, the focus on youth athletes limits the generalizability of the findings to other age groups or non-competitive athletes. While bibliometric analysis identifies patterns in publications and unknowns by subject, the quality of studies and interventions is not judged. Systematic reviews and/or meta-analyses in the future might complement this study by critiquing methodology and interventions’ effectiveness in ongoing studies.

Despite these limitations, this study offers valuable insights into the current state and future directions of research on the mental health of young athletes. As mental health continues to gain attention in the realm of sports, our findings underscore the need for more interdisciplinary research and targeted interventions to support the psychological well-being of athletes. By addressing these challenges, stakeholders can contribute to creating a healthier and more supportive environment for youth athletes. Sports organizations ought to incorporate psychological health education in sailing programs, and provide their trainers and instructors with tools to recognize and guide youth sailors who exhibit psychological distress. Psychological resilience strategies, including mindfulness and cognitive behavioral interventions, may improve young competitors’ ability to handle challenging situations.

## 5. Conclusions

In conclusion, this study provides a comprehensive bibliometric analysis of the state of research on youth sailors and mental health, using data from the Web of Science (WOS) database from 2014 to 2024. The analysis emphasizes that increasing attention has been directed towards understanding the mental health of youth sailors throughout this period, indicating a burgeoning interest among researchers and practitioners alike. Despite the limitation of relying solely on the WOS database, which may not encompass the entirety of relevant journal literature, the key issues in youth sailors’ mental health research appear to remain consistent, highlighting the need for sustained focus in this area. Future studies could greatly benefit from incorporating additional databases to ensure a broader and more comprehensive scope, allowing for a detailed understanding of the multidisciplinary facets that impact young sailors’ mental health.

Countries such as Canada, the USA, Australia, and China have been at the forefront of research efforts in this field, demonstrating that despite geographical and cultural differences, youth sailors across various nations face comparable mental health challenges, including depression, anxiety, and various psychological disorders. This global pattern suggests that mental health issues among young sailors are universal phenomena, transcending specific cultures or regions and thereby indicating a pressing need for international collaboration in addressing these challenges. Such insights could pave the way for shared interventions and best practices that can be adapted across different cultural contexts.

Current research has shown a concentrated focus on key topics such as depression, anxiety, and disorders, reflecting a growing awareness of the critical importance of addressing these issues within the sports context. As mental health increasingly becomes an essential aspect of athletic performance—especially in the demanding environments associated with competitive sailing—it is essential that coaches, families, and researchers collaborate closely to support the psychological well-being of young athletes. The challenge lies in devising effective strategies that can encourage mental resilience from the early stages of training, guaranteeing that these athletes are equipped to handle the pressures they face both during competition and in their everyday lives.

This study presents an extensive bibliometric profile of youth athletic mental health research, including prominent trends, top authors, and emerging areas of inquiry. The insights derived are crucial for health professionals, sporting policymakers, and sport psychologists in designing evidence-based interventions for youth athletes. Future research should prioritize collaborative, transdisciplinary collaborations between health professionals and sporting institutions to facilitate incorporation of mental health services in athletic training. Bridging clinical application and scientific studies, these interventions hold potential to enhance youth athletic psychological health. As this analysis is only conducted on WOS, publications from databases such as Scopus and PubMed, if any, might have been overlooked. Future studies should cover several databases for comprehensive bibliometric assessment.

## Figures and Tables

**Table 1:** Data retrieval summary for the manuscript.

Category	Specific Standard Requirements
Research Database	Web of Science core collection
Citation Indexes	SCI-EXPANDED, SSCI
Searching Period	2014–2024
Language	English
Searching Keywords	“youth mental health, sport”, “youth mental health, sailors”, “youth mental health, athletes”
Document Types	All
Samole Size	1296

**Table 2:** Keywords used in literature search from WOS and search results.

No.	Document Type	Count
1	“youth mental health, sport”, “youth mental health, sailors”, “youth mental health, athletes”	1296
2	Original Articles	1084
3	Review Article	165
4	Early Access	39
5	Meeting Abstract	24
6	Editorial Material	19
7	Retracted Publication	7
8	Letter	3
9	Proceeding Paper	2
10	Correction	1
11	Finalised articles	315

**Table 3:** Journal partition.

Zone	Publication/Journal	Number of Journals	Number of Publications
First Zone	6≥	8	84
Second Zone	3–5	21	86
Third Zone	1–2	114	145

**Table 4:** Top five high paper productivity countries/region.

Rank	Country/Region	Documents	Citations	Average Citation
1	Australia	97	3065	31.60
2	USA	84	2042	24.30
3	Canada	64	2240	35
4	England	36	1204	33.44
5	China	30	1018	33.93

**Table 5:** Top 10 relevant research organisations.

Rank	Organization	Documents	Citations	Average Citation
1	Univ Melbourne	46	1036	22.52
2	Orygen	19	335	17.63
3	Deakin Univ	16	155	9.6875
4	Univ Wollongong	16	1036	64.75
5	Victoria Univ	15	175	11.67

**Table 6:** Ranking of significant influential authors in this research area.

Rank	Author	Documents	Citations	Average Citation
1	Purcell, Rosemary	20	584	29.2
2	Rice, Simon	16	252	15.75
3	Swann, Christian	10	347	34.7
4	Vella, Stewart A.	12	419	34.92
5	Reardon, Claudia I.	6	219	36.5

**Table 7:** Top 3 ranking of journals publishing the most relevant articles.

Rank	Source	Publications	Citations	Average Citation
1	*Frontiers in Psychology*	17	236	14
2	*Psychology of Sport and Exercise*	17	238	13.88
3	*International Journal of Research and Public Health*	15	505	33.67

**Table 8:** Keyword frequency ranking using bibliometric analysis.

Rank	Keyword	Frequency	Total Link Strength
1	Mental Health	145	353
2	Physical Activity	89	218
3	Youth	89	208
4	Children	82	207
5	Depression	76	198

## Data Availability

More data will be provided on request to the corresponding author.
